# Oncologic Therapy Support Via Means of a Dedicated Mobile App (OPTIMISE-1): Protocol for a Prospective Pilot Trial

**DOI:** 10.2196/resprot.8915

**Published:** 2018-03-06

**Authors:** Rami A El Shafie, Nina Bougatf, Tanja Sprave, Dorothea Weber, Dieter Oetzel, Timo Machmer, Peter E Huber, Jürgen Debus, Nils H Nicolay

**Affiliations:** ^1^ Department of Radiation Oncology Heidelberg University Hospital Heidelberg Germany; ^2^ Heidelberg Institute for Radiation Oncology Heidelberg Germany; ^3^ Heidelberg Ion Beam Therapy Center Heidelberg Germany; ^4^ Institute for Medical Biometry and Informatics University of Heidelberg Heidelberg Germany; ^5^ OPASCA GmbH Mannheim Germany; ^6^ Department of Radiation Oncology German Cancer Research Center Heidelberg Germany; ^7^ Department of Radiation Oncology Freiburg University Medical Center Freiburg Germany

**Keywords:** mHealth, radiotherapy, mobile application, quality of life, cancer, mhealth, Patient-Reported Outcome Measures (PROMs)

## Abstract

**Background:**

The increasing role of consumer electronics and Web-enabled mobile devices in the medical sector opens up promising possibilities for integrating novel technical solutions into therapy and patient support for oncologic illnesses. A recent survey carried out at Heidelberg University Hospital suggested a high acceptance among patients for an additional approach to patient care during radiotherapy based on patient-reported outcomes by a dedicated mobile app.

**Objective:**

The aim of this trial (OPTIMISE-1: Oncologic Therapy Support Via Means of a Dedicated Mobile App – A Prospective Feasibility Evaluation) is to prospectively evaluate the feasibility of employing a mobile app for the systematic support of radiooncological patients throughout the course of their radiotherapy by monitoring symptoms and patient performance, and facilitating the background-exchange of relevant information between patient and physician.

**Methods:**

The present single-center, prospective, exploratory trial, conducted at Heidelberg University Hospital, assesses the feasibility of integrating an app-based approach into patient-care during radiotherapy. Patients undergoing curative radiotherapy for thoracic or pelvic tumors will be surveyed regarding general performance, treatment-related quality of life (QoL) and symptoms, and their need to personally consult a physician by means of a mobile app during treatment. The primary endpoint of feasibility will be reached when 80% of the patients have successfully answered 80% of their respective questions scheduled for each treatment day. Furthermore, treatment-related patient satisfaction and health-related QoL is assessed by the Patient Satisfaction Questionnaire Short Form (PSQ-18) and the European Organization for Research and Treatment of Cancer (EORTC) questionnaires at the beginning (baseline) and end of radiotherapy, and at the first follow-up.

**Results:**

This trial will recruit 50 patients over a period of 12 months. Follow-up will be completed after 18 months, and publication of results is planned at 24 months after trial initiation.

**Conclusions:**

This study will serve as a basis for future studies aiming to exploit the constant innovation in mobile medical appliances and integrate novel patient-centered concepts into patient care in the context of radiotherapy.

**Trial Registration:**

ClinicalTrials.gov NCT03168048; https://clinicaltrials.gov/ct2/show/NCT03168048 (Archived at WebCite http://www.webcitation.org/6wtWGgi0X)

## Introduction

The role of consumer electronics and Web-enabled mobile devices in the medical sector is ever increasing, broadening the scope for mobile health apps [1]. The availability and market share of such appliances, summarized by the World Health Organization under the term “mobile health” (mHealth), has steadily grown over the past years [2]. Areas of use include assistance in appointment-making, the monitoring of certain parameters in patients with chronic illnesses or the regular documentation of pain or other symptoms [3-5]. Initial experience for the use of mobile apps in the area of oncology has shown promising results: A prospective pilot trial was able to demonstrate significantly improved overall survival for lung cancer patients, who were systematically telemonitored with the help of a mobile phone app based on patient-reported data and using the dynamics of patients’ clinical symptoms for risk-stratification and individualized follow-up [6]. This study showed that the use of an app-based identification of high-risk patients may help to initiate earlier treatment, contributing to improved patient outcomes. Furthermore, the patient compliance within the scope of a systematic follow-up regimen proved superior to the control group whose follow-up only consisted of regular computed tomography scans and no additional supervision by a mobile phone–based app [7].

On the other hand, a survey among German health care providers showed a high readiness to incorporate the use of mobile health apps into oncologic treatment regimens, thereby utilizing the obvious advantages of mobile devices in patient care [8]. In this survey, the ability to monitor patient performance more closely even over longer geographical distances, the possibility to overcome language barriers or selectively address special needs of patient subgroups, such as ethnic minorities or children, were reported among the main advantages of mHealth in the field of oncology [9,10].

At present, the spectrum of apps available in the oncologic field is still rather limited and lacks validation by larger systematic analyses, so that the relevance of mobile apps for patient care and therapy support during prolonged oncologic treatments, such as radiotherapy or chemotherapy, remains largely unclear [11].

Based on the promising preliminary results of a systematic survey being conducted at Heidelberg University Hospital among a large number of cancer patients demonstrating a high acceptance of mobile app–based measures of therapy support, the present OPTIMISE-1 trial (which stands for **O**ncologic Thera**p**y Suppor**t** V**i**a **M**eans of a Ded**i**cated Mobile App – A Pro**s**pective F**e**asibility Evaluation) aims to assess the feasibility of introducing a mobile app to the treatment schedule of patients undergoing radiotherapy, as well as measuring its impact on patient satisfaction and health-related quality of life (QoL).

## Methods

### Study Design

The present study is designed as a prospective, single-center pilot study and will be conducted at Heidelberg University Hospital’s Department of Radiation Oncology. Cancer patients undergoing radiotherapy are offered a novel model of therapy support during the course of treatment, where a mobile app will be used to monitor patient-reported outcomes concerning general performance and therapy-related symptoms, and enable patients to express the need to see a physician when clinical symptoms occur. The length and frequency of consultations with a physician will be analyzed to assess the economic aspect of the model. Furthermore, the necessity of admitting a patient to inpatient care during the course of therapy will be documented together with the timepoint of admission. Since routine bloodwork is independently conducted at regular intervals for all patients undergoing radiotherapy, the data from this bloodwork together with the clinical parameters documented in this study (eg, symptoms, toxicity, etc) will be screened for predictive factors for the necessity of inpatient admission by means of correlation. This is done as part of an exploratory side analysis unrelated to the primary objective of this study.

### Recruitment

Patients are screened for eligibility during their first consultation at our department prior to radiotherapy by a study nurse. The consulting radiation oncologist presents each eligible patient with information about the trial and answers any questions about its nature, course, benefits and risks. If radiotherapy is medically indicated and the patient provides informed consent, the patient is included in the trial. Relying on logistic considerations based on the expected annual recruitment of patients fulfilling the inclusion criteria, the recruitment period has been set at 12 months. Roughly 1000 patients are available for screening yearly, based on intrainstitutional patient statistics. This means that the likelihood of fulfilling the inclusion criteria is highly probable. On the basis of a conservative assumed consent ratio of 5%-10%, meeting the recruitment target within 12 months is realistic.

**Figure 1:**
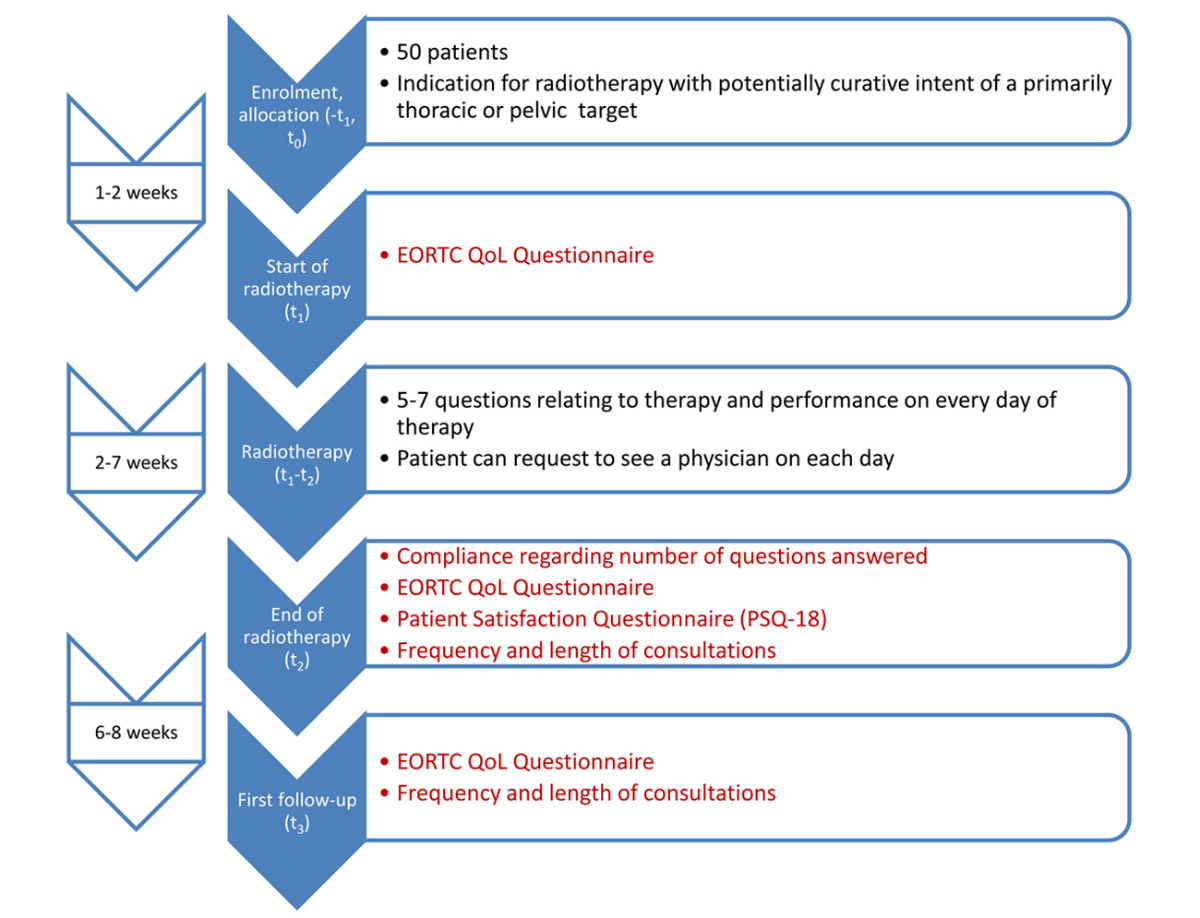
Time-line for trial subjects. Endpoints are marked in red. EORTC QoL questionnaire: European Organisation for Research and Treatment of Cancer quality of life questionnaire.

The study duration for each individual subject depends on the duration of radiotherapy, which differs with diagnosis. Typically, the period from the start of radiotherapy until the first follow-up will be between 10 and 14 weeks. A detailed timeline with interventions and assessments is illustrated in Figure 1.

### Target Population

The study population includes patients receiving radiotherapy of a primarily thoracic or pelvic target and with a curative intent. This applies to a range of malignancies and depending on diagnosis to a range of tumor stages. This approach is intended to diversify the sample on which the mobile app is tested. Examples for thoracic targets are cancers of breast and lung, whereas pelvic targets include malignancies of uterus, vagina and vulva, prostate, and rectum.

Patients receiving a regime of chemoradiation that prospectively requires them to be treated as inpatients for a substantial portion of the duration of radiotherapy are not eligible to participate in this trial. According to standard operating procedures (SOP) at our institution, this would exclude most patients with cancers of the cervix, esophagus, anus and rectum. Exceptions from this rule are possible in individual cases (eg, patients with rectal cancer, very good clinical performance and oral chemotherapy), if the screening physician deems it feasible to conduct the treatment on an outpatient basis.

### Inclusion and Exclusion Criteria

Patients enrolled in this pilot trial must fulfill the following inclusion criteria:

- Indication for radiotherapy with potentially curative intent of a primarily thoracic or pelvic target
- Karnofsky performance score ≥70%
- Prospective ability to receive treatment as an outpatient
- ≥18 years of age

Patients with the inability to give informed consent will be excluded from the study.

### App-Based Therapy Support

**Figure 2:**
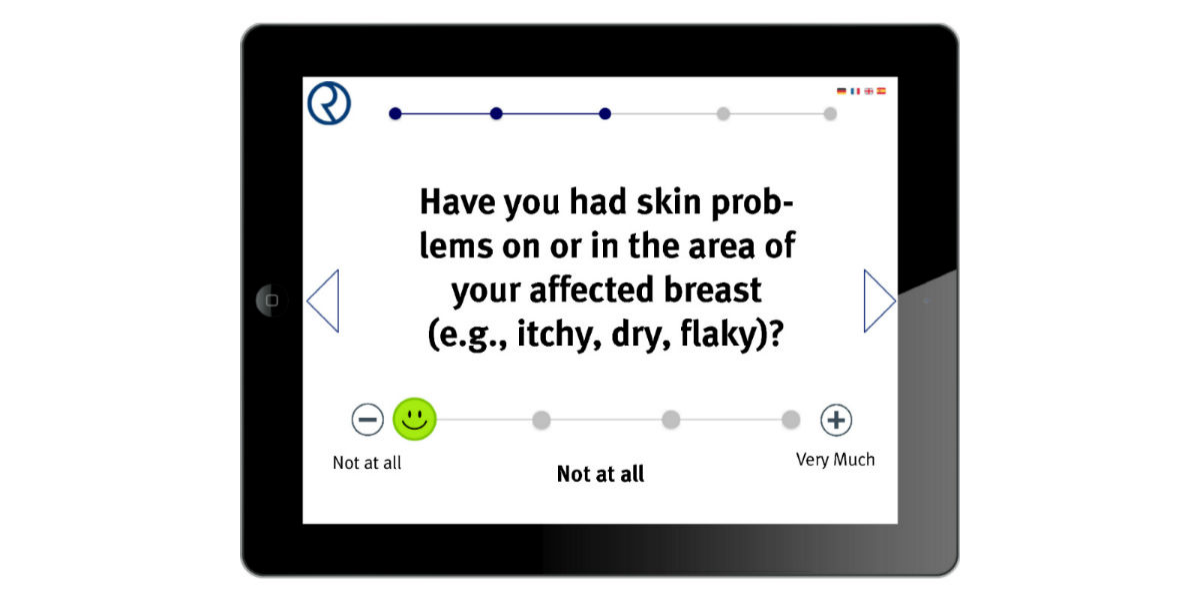
Sample screenshot of the mobile application for patient support during radiotherapy.

Patients will be given a mobile tablet device and will be asked to answer 5-7 differing questions. The respective answers will be collected as patient-reported outcome. This type of digital survey is conducted during clinic hours on each day prior to radiotherapy. A sample screenshot is displayed in Figure 2.

To assess the patient’s general and specific wellbeing during radiotherapy, a system of rotating questions based on the European Organisation for Research and Treatment of Cancer (EORTC) quality of life questionnaires (QLQ) has been developed. Since the wording of the questions is extracted directly from the EORTC questionnaires, which are available in multiple languages, the OPTIMISE-1 survey app will be multilingual. Depending on individual diagnosis, the patient is presented with questions extracted from the respective EORTC module (eg, BR23 for breast cancer, PR25 for prostate cancer, LC13 for lung cancer; all modules available on the EORTC website) that cover the patient’s therapy-related wellbeing, symptoms for expected toxicities and general performance. Questions are automatically rotated by the application so that by posing 5-7 questions daily, the complete module is covered within one week of therapy. A diagnosis-dependent questionnaire rotation schedule is assigned to each patient prior to the beginning of radiotherapy with the help of a Web-based backend interface. In addition to the aforementioned rotating questions, unchanging questions are posed every day at the beginning and end of the survey.

For example, patients are asked to describe their general health/QoL subjectively on a 7-point scale covering a range from “excellent” to “very poor” (adapted from EORTC QLQ-C30). Furthermore, patients are asked to state whether or not they feel the need to personally consult a physician on that respective treatment day. If they do, upon completing the survey, an automatic notification is triggered at the medical-technical assistants’ management terminal, prompting them to schedule a visit with the supervising physician on that day. The information provided via the survey application is presented regularly to a supervising physician via the Web-based backend interface used for monitoring treatment and toxicity and preparing for regular patient visits. Routine check-up visits are conducted once weekly, and additional visits are arranged as requested by the patients via the app. The number and length of all visits are documented. The app-based therapy support is continued daily as long as the patient is in outpatient care.

The standard of care outside this trial at our institution for patients undergoing outpatient radiotherapy consists of weekly scheduled visits with a supervising physician and bloodwork at an interval depending on diagnosis (usually once weekly or less). The therapy support provided within this trial represents an additional, more close-knit and individualized form of care during radiotherapy.

### Assessment of Primary and Secondary Endpoints

The primary aim of this study is to assess the feasibility of an app-based therapy support. It will be assessed by the primary endpoint—the proportion of patients who answered at least 80% of the app-based questions during their radiotherapy as outpatients. For every study subject, the number of answered questions is documented on each day of radiotherapy for the assessment of the primary endpoint. This definition of feasibility was derived mainly from clinical and logistical considerations. Due to logistical difficulties unrelated to the trial (eg, personnel change, transfer of patients to different radiation units due to technical maintenance), a certain portion of treatment days is to be expected where some patients fail to receive the survey tablet device. Since there is no previous systematic data on this specific issue, we estimate the portion of those expected irregularities at a realistic 20% based on clinical experience.

The following secondary endpoints are considered:

- Patient satisfaction
- Number of medical consultations
- Duration of medical consultations (in min)
- Need for toxicity-caused hospitalization
- Health-related QoL

Patient satisfaction is measured by the PSQ-18, which includes 18 items in 7 subscales: general satisfaction, technical quality, interpersonal manner, communication, early reaction to treatment side effects, time spent with doctor, accessibility and convenience. Responses to each item are given on a 5-point scale, and based on the questionnaire, a total sum score will be calculated and transformed to a scale ranging from 1 to 5.

Health-related QoL is assessed by the EORTC QLQ-C30 questionnaire in addition to the diagnosis-specific questionnaire module (eg, BR23 for breast cancer or PR25 for prostate cancer). Patients are prompted to fill out these questionnaires prior to the initiation of radiotherapy, at the time of treatment completion and at the first follow-up visit, which is routinely scheduled at 6-8 weeks after the completion of therapy. The duration and number of all visits during radiotherapy is documented to estimate the average cost of this form of therapy support. Per our SOP, the beginning and end of every consultation for all patients is documented by the physician by setting status flags within the Radiation Oncology Clinic Information System (ROKIS). The duration of every consultation can then be extracted by querying the ROKIS database. The software solutions in use for those purposes at our institution are MOSAIQ (ELEKTA, Stockholm, Sweden) for ROKIS and Crystal Reports (SAP, Walldorf, Germany) for generating database reports.

Table 1 shows the intervention and assessment schedule for the trial. The Standard Protocol Items: Recommendations for Interventional Trials (SPIRIT) checklist [12] on different protocol items related to this trial is included in Multimedia Appendix 1.

### Statistical Analysis

The calculation of the sample size was done with respect to the primary endpoint and the primary analysis. Using the exact binomial test, we aim to show that the primary endpoint is reached at values of ≥80% of all study subjects answering ≥80% of the app-based questions during their radiotherapy as outpatients. With a sample size of 50 patients in this pilot study and an assumed percentage of 92% for the primary endpoint, a power of 1-β=80% is reached with a one-sided significance level of α=5%. Considerations regarding sample size and power were done using the *PROC POWER* procedure in SAS (version 9.4). The primary endpoint will be evaluated using the exact binomial test, which is the primary analysis. The one-sided significance level for this feasibility study is set to 5%.

Methods of descriptive data analysis will be used to describe patient characteristics and secondary outcomes, including calculation of appropriate summary measures of the empirical distribution, such as mean and standard deviation, median and interquartile range for continous variables, as well as absolute and relative numbers for categorical variables. Additionally, according to tumor type, subgroup analyses will be performed as a sensitivity analysis. Additionally, multivariable regression models will be used to analyze the relationship between patient characteristics and the primary endpoint. However, we will consider the primary variable on the original scale without any cut-off value (ie, the relative number of app-based answered questions) as the outcome variable and, thus, linear regression models will be applied. Age, gender and the need of toxicity-caused hospitalization will be included as predictors. The same model will be applied for the secondary endpoints. In addition, correlations will be considered. Graphical methods will be applied to visualize the results.

### Ethics Approval and Informed Consent

The Heidelberg Ethics Committee approved this study on June 26, 2017 (S-216/2017). Inclusion of a patient into this trial requires meeting the above-mentioned inclusion and exclusion criteria as well as the patient’s written informed consent. Continuous information will be supplied to the committee about all changes to the study that may influence patient safety, as well as about the conclusion or discontinuation of the study. Participation in the clinical trial is voluntary for subjects. Before inclusion in the study, a potential subject will be thoroughly and in detail informed about the nature, aims, risks and benefits of the study before informed consent can be given. Detailed information will be provided in a fashion and language understood by the patient.

An informational handout as well as an informed consent form—both documents conforming to the standards of the International Conference on Harmonization-Good Clinical Practice—will be provided to the patient before inclusion. Informed consent must be given only after an appropriate amount of time for consideration and then must be in writing and complemented with information about date and time of signature in the patient’s own handwriting. Informed consent must be countersigned by the treating physician. If a patient is incapable of signing the informed consent form, oral informed consent must be confirmed by the signature of a witness.

Clinical subjects are free to refuse to participate in the study or to withdraw from it at any time for any reason without incurring any penalty or withholding of treatment on the part of the investigator. This study includes no additional invasive or otherwise harmful or burdening procedures.

**Table 1:** Intervention and assessment schedule for the OPTIMISE-1 trial.

Steps		Enrolment	Allocation	Start of RT^a^	End of RT	First follow-up
**Enrolment**						
	Eligibility screen	✓				
	Informed consent	✓				
	Allocation		✓			
**Interventions**						
	RT			✓	✓	
	Patient support with help of a mobile app			✓	✓	
**Assessments**						
	EORTC QLQ^b^			✓	✓	✓
	# of app-based questions answered				✓	
	Patient satisfaction (PSQ-18^c^)				✓	
	Frequency and length of consultations				✓	✓

^a^RT: radiotherapy.

^b^EORTC QLQ: European Organisation for Research and Treatment of Cancer quality of life questionnaires.

^c^PSQ-18: Patient Satisfaction Questionnaire Short Form.

## Results

The results of this trial will be published in a peer-reviewed journal within 24 months of trial initiation, independently of the outcome of the trial. Publication will be prepared under the lead of the principal investigator of the study. The first and last authorship will be assigned by the principal investigator.

## Discussion

### Study Rationale

As consumer electronics become increasingly employed in the medical sector, their use for the support of oncologic patients opens up a range of possibilities from the gathering of information on patient health and symptoms to patient notification about appointments or test results. The present single-center prospective pilot trial conducted at the Department of Radiation Oncology of Heidelberg University Hospital explores the possibility of employing a mobile app for the systematic support of radiooncological patients throughout the course of their radiotherapy by monitoring symptoms and patient performance, and facilitating the exchange of relevant information between patient and physician. We aim to assess the feasibility and patient acceptance of incorporating such a mobile app into active patient care. Furthermore, we aim to estimate the economic and financial aspects of this novel approach to therapy support.

### Limitations and Future Perspectives

In the current study, the survey app is not deployed using the patient’s own mobile device but is installed on a tablet computer belonging to the investigator and integrated into the institution’s information technology infrastructure according to data protection regulations. For future trials, it is planned (after establishing feasibility and usefulness) to expand the scope of the app so that patients can use their own devices for survey completion outside the clinic.

On the other side, the aforementioned approach may contribute to addressing a possible selection bias. By presenting the application to patients on an institution-owned tablet device, we do not exclude patients who do not themselves own a mobile device, thus testing the application on patients regardless of their technical abilities and/or affinity.

The definition of feasibility in this trial is largely derived from intrainstitutional clinical experience and logistical considerations. There is, however, to date no systematic data from which a more general definition of feasibility can be derived in this context. A systematic survey conducted at our institution on oncologic patients’ use of and affinity towards mobile devices is in accordance with previously published data [13] in suggesting that roughly three quarters of patients would be inclined to use a mobile app in the context of radiotherapy. This further suggests that expecting a patient compliance of 80% to demonstrate feasibility is realistic.

### Conclusions

The findings of this study will play an important role in clinically validating the mobile app we have developed for the collection of patient-reported outcome measures from patients undergoing radiotherapy. By proving the usefulness and feasibility of an app-based approach to radio-oncologic patient supportive care, the present exploratory study prepares the ground for the larger-scale evaluations we are planning to perform on this promising subject.

## Appendix

Multimedia Appendix 1 SPIRIT checklist of different protocol items related to this trial.

## Abbreviations

EORTC: European Organisation for Research and Treatment of Cancer
mHealth: mobile health
OPTIMISE-1: Oncologic Therapy Support Via Means of a Dedicated Mobile App – A Prospective Feasibility Evaluation
PSQ-18: Patient Satisfaction Questionnaire Short Form
QLQ: quality of life questionnaire
QoL: quality of life
ROKIS: Radiation Oncology Clinic Information System
SOP: standard operating procedures
SPIRIT: Standard Protocol Items: Recommendations for Interventional Trials
